# A systematic review of suicide risk management strategies in primary care settings

**DOI:** 10.3389/fpsyt.2024.1440738

**Published:** 2024-09-02

**Authors:** Monika Sreeja Thangada, Rahul Kasoju

**Affiliations:** ^1^ Department of Psychiatry, Harry S Truman Veterans Hospital, Columbia, MO, United States; ^2^ Department of General Medicine, Sri Venkata Sai (SVS) Medical College, Mahbubnagar, TG, India

**Keywords:** primary care, general practice, suicidal risk factors, suicide risk management, reducing suicide rates, improving suicide prevention efforts, suicide prevention, suicide attempts

## Abstract

**Introduction and Objective:**

Suicide is a major public health concern. Recently, suicide rates have increased among traditionally low-risk groups (e.g., white, middle-aged males). Suicide risk assessments and prevention strategies should be tailored to specific at-risk populations. This systematic review examines suicide risk detection and management in primary care, focusing on treatments to reduce suicide rates and improve prevention efforts.

**Methodology:**

A systematic review was conducted following PRISMA guidelines. Literature was collected and analyzed using Boolean operators with relevant keywords in databases (e.g., PubMed, Google Scholar, PsycINFO) to identify randomized and non-randomized studies focusing on suicide risk factors and management strategies in primary care, published in the past 10 years. The risk of bias 2.0 and Newcastle Ottawa scale was used to assess risk of bias, and data from moderate-quality studies were synthesized.

**Results:**

Thirteen moderate-quality studies were reviewed. Key findings include the need for assessing modifiable risk factors like substance use and mental health. General practitioner (GP) engagement post-suicide attempt (SA) improves outcomes and reduces repeat SAs. Effective strategies include comprehensive risk assessments, collaborative treatment, and enhanced GP support. Barriers to effective suicide prevention include insufficient information, judgmental communication, lack of positive therapeutic relationships, and inadequate holistic assessments. These findings highlight the need for tailored suicide prevention strategies in primary care. However, the evidence sample size is small with reduced statistical power that limits generalizability. The included studies were also regional examinations, which restrict their broader relevance.

**Discussion:**

Significant risk factors, barriers, and effective strategies for suicide prevention were identified. For children aged 12 or younger, preexisting psychiatric, developmental, or behavioral disorders, impulsive behaviors, aggressiveness, and significant stressful life events within the family were critical. For adults, loneliness, gaps in depression treatment, and social factors are significant. Barriers to suicide prevention included insufficient information, judgmental communication, lack of positive therapeutic relationships, inadequate holistic risk assessments, lack of individualized care, insufficient tangible support and resources, inconsistent follow-up procedures, variability in risk assessment, poor communication, stigma, and negative attitudes. Effective methods include the Postvention Assisting Bereaved by Suicide training program, continued education, comprehensive clinical assessments, individualized care, and community-based interventions like the SUPRANET program.

**Systematic Review Registration:**

https://www.crd.york.ac.uk/PROSPERO, identifier CRD42024550904.

## Introduction

Suicide is a significant public health issue. Between 2008 and 2019, suicide rates in the United States increased from 481 to 564 per 100,000 in primary care populations (1). Although overall rates have risen, they have decreased in some age groups. While suicide prevention strategies targeting high-risk subpopulations have received significant attention, rates have also increased in traditionally low-risk demographic groups, such as white, middle-aged men (1). Suicide risk assessments and preventive activities should be customized to distinctly high-risk populations (2).

General practitioners (GPs), known as primary care providers (PCPs) in the US healthcare system, play a crucial role in the early identification and management of suicidal risk factors (3). GPs/PCPs often serve as the first point of contact for patients within the healthcare system, providing continuous and comprehensive care, including mental health assessment and intervention (4). This review aims to explore the existing literature regarding the identification of risk factors and the management of suicide in primary care settings, focusing on interventions aimed at reducing suicide rates and improving prevention efforts. The goal is to inform clinical practice and guide future research in this critical area.

Suicide is often linked to psychiatric problems and has multiple causes. Clinical patient groups have a four-fold higher lifetime suicide risk compared with the general population (5). Patients with certain psychiatric diseases, such as major depressive disorder, can have lifetime risk rates up to 20 times higher (6). Suicide risk is also increased among patients with specific medical conditions, psychiatric disorders, and acute psychiatric symptoms (7). Early life sexual abuse, domestic violence among married women, and partner violence are additional risk factors that may lead to suicide attempts (8, 9). Consequently, psychiatrists and other medical specialists must recognize and respond to suicidal ideation in their clinical practices.

One-third of Americans and one-fourth of Britons seek mental health care in the year before suicide (10–12). Approximately 10% of those who die by suicide had visited an emergency department (ED) within the preceding two months (13). An estimated 31.3% of Americans who died by suicide had received mental health care (14). Suicide is one of the five most commonly reported sentinel events in hospitals, resulting in significant injury or death (15). Over 80% of suicide deaths in reported sentinel episodes are attributed to inadequate patient assessment (16).

Clinicians can prevent suicide by identifying and treating at-risk patients. However, academic training for psychiatry students has not kept up with developments in suicide risk assessment. A psychiatrist’s ability to assess suicide risk is essential for providing effective therapy and care (17). Standard treatment for patients at suicide risk requires psychiatrists to conduct appropriate suicide risk assessments (18–20). However, psychiatrists sometimes lack the necessary expertise to screen for suicide risk accurately (21, 22). Surprisingly, individuals who died by suicide were more likely to have been deemed low risk in their previous assessment (23). Therefore, it is necessary to be familiar with the concepts of suicidality (24) (Suicidality is the state of being at risk of committing suicide, typically characterized by thoughts or intentions of suicide, particularly when accompanied by an elaborate plan), self-harm (25) (When someone injure itself on purpose to hurt without causing death), and suicide attempt (26) (A suicide attempt refers to the deliberate self-inflicted harm by an individual with the intention of ending their life, although they do not succeed in causing their own death) to identify them as risk factors and devise management strategies.

There are crucial aspects of suicide management in primary care. Studies highlight the importance of integrating behavioral health into primary care to improve suicide prevention (27). However, primary care settings often lack consistent follow-up for adolescents with suicide concerns due to depressive symptoms, indicating a need for national guidelines to enhance practices (28). Research in Uganda emphasizes primary healthcare workers’ challenges in assessing and managing suicidality, stressing the importance of improving their knowledge and attitudes for equitable services (29). Supporting GPs in suicide risk assessment among young people and improving clinical decision-making can be achieved through educational training and continued education (30).

Despite existing research, there is a lack of systematic reviews that summarize available evidence on risk factors and management in primary care. Managing risk factors and improving management approaches in general practice involve a multi-disciplinary, knowledgeable, and resourceful behavioral health system. According to Richards et al. (2019), integrating Behavioral Health services in primary care, with an emphasis on depression screening and suicide risk evaluation, showed that primary care personnel value the ability to provide necessary care for suicidal patients. Integrated social workers, trained as behavioral health clinicians, along with psychiatrist leadership and consultative assistance, offer a comprehensive approach to assist primary care teams in achieving success (31).

Thus, this systematic review aims to explore the existing literature regarding identifying risk factors and managing suicide in primary care settings, focusing on interventions aimed at reducing suicide rates and improving prevention efforts. The goal is to inform clinical practice and guide future research.

## Materials and methods

This systematic review followed the Preferred Reporting Items for Systematic Reviews and Meta-analysis (PRISMA) statement (32). A protocol was registered prospectively with PROSPERO, Centre for Reviews and Dissemination, University of York: CRD42024550904.

### Study design

This systematic review is based on analyzing screened studies related to identifying and managing suicide risk factors in primary care settings.

### Search strategy

This systematic evidence-based practice used the Patient, Problem, Population, Intervention, Comparison, Outcome (PICO) framework to generate specific, answered research questions. The following PICOS framework was applied: Population/problem—patients in primary care settings; Primary care; General Practice; Primary healthcare prevention; “Primary health care” [MeSH]; Intervention/Exposure—identification and management of suicide risk factors; Suicide risk management; Suicide risk factors; “Risk factors” [MeSH], “Risk management” [MeSH]; Comparison—none/standard care; and Outcomes—reduction in suicide rates; improving suicide prevention efforts; Reducing suicide rates; “Suicide prevention” [MeSH], “Suicide, attempted” [MeSH]. The systematic review was conducted to search literature using Boolean operators with relevant keywords, and medical subject headings (MeSH) mentioned earlier in databases, e.g., PubMed, PsycINFO and Google Scholar, to retrieve open access, full text available, English language, last ten years (2014–2024) studies. The Mesh words and keywords were mentioned in the **Supplementary Table 1**.

### Research question

What are the methods for the identification and management of suicide risk factors to reduce suicide rates and enhance suicide prevention efforts in primary care settings?

### Studies selection criteria

#### Inclusion criteria

Included studies: were performed in primary care settings; had the stated objective of identifying or managing suicide risk factors; included patients of all age groups and genders; included review-related outcomes.

Quantitative (randomized controlled, prospective cohort, retrospective observational) and qualitative (survey) studies relevant to the review objective were considered. Studies available in full text were also considered and analyzed. Only English-language studies were included. Only studies conducted within the last 10 years were included to focus on direct relevance to current practice.

#### Exclusion criteria

**Figure 1:**
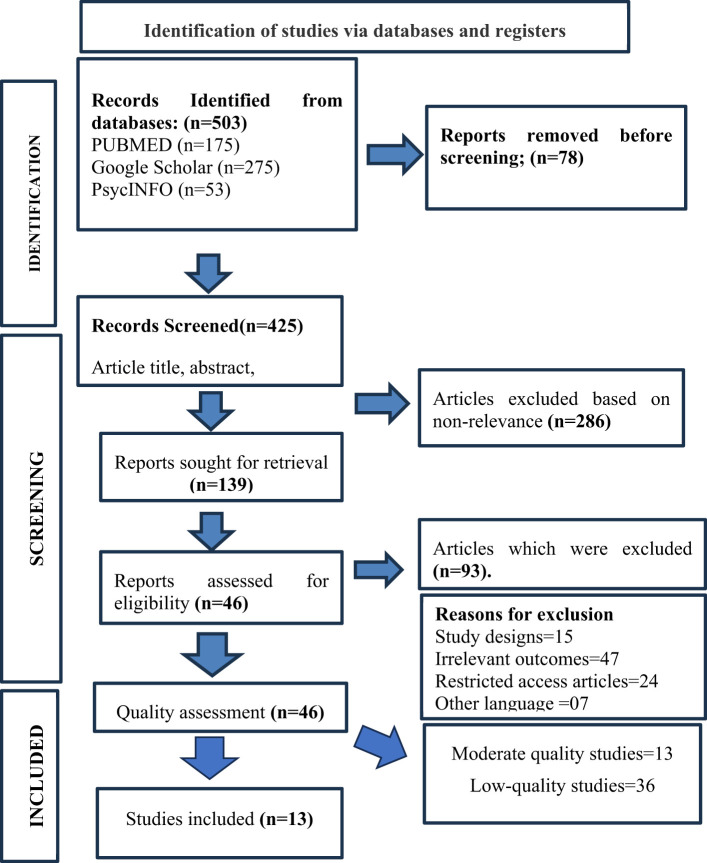
PRISMA Flow Chart.

Studies were excluded if they were case reports, case series, or review articles conducted in secondary or tertiary care settings; articles paid and their full text is inaccessible; articles with unclear or insufficient methodological descriptions; articles published before 2014; articles that did not address specific suicide risk factor-related assessment or prevention; or articles written in a language other than English. The reasons for the excluded studies are mentioned in **Figure 1**.

### Studies selection process

The study selection process followed the PRISMA guidelines. Initially, 503 studies were identified, and 78 duplications were removed using Endnote X9, with the remaining 425 undergoing screening. Screening, abstract titles, and in-depth reading will exclude 286 irrelevant studies. The eligibility status of the remaining 139 studies was checked, and 46 studies met inclusion criteria, whose quality assessment was performed (**Figure 1**).

### Quality assessment

To determine evidence certainty, Risk of bias 2.0 was used for one identified RCT, and 45 Non-randomized studies were assessed using the Newcastle-Ottawa Scale (NOS) (33, 34). The risk of bias evaluates six domains: randomization, bias due to deviation from intended intervention, missing outcome data, measurement of outcome, and bias in the selection of reported results with overall bias (35). The NOS score considers the selection of study groups, comparability of groups, and ascertainment of exposure/outcome. A score was assigned based on these criteria. The researcher assessed the risk of bias, methodology, results consistency, and limitations using the score scale. The NOS was used to categorize studies as having a high (score ≥7), moderate (score 5 or 6), or low (score ≤4) risk of bias. Higher NOS scores thus indicate better methodological quality and greater confidence in the study findings. This tool assesses data reliability and validity to ensure informed-based decision-making in evidence-based practice. In addition, consistency across study findings, the magnitudes of their effects, and the relevance of their findings to the research question were considered during the quality assessment (36) (**Supplementary Table 2**).

### Strength of recommendation

The randomized controlled trial was rated using the GRADE (Grading of Recommendation, Assessment, Development, and Evaluation) approach, which assigned rating as high risk of bias into low quality, uncertain risk of bias into moderate quality, and low risk of bias into high quality depending upon the six domains (Study design, risk of bias, inconsistency, indirectness, imprecision, publication bias) (37). The NOS score was used to classify the strength of recommendation using (38) for each included study, where a NOS score ≥7 indicated high-quality evidence that was considered to provide high-strength recommendations; a NOS score of 5 or 6 indicated moderate-quality evidence that was considered to provide moderate-strength recommendations; and NOS score ≤4 indicated low-quality evidence that was considered to provide weak recommendations. At last, 13 studies of moderate quality, one RCT and 12 non-RCTs, were included in this review (**Supplementary Table 3**), while the remaining 36 low-quality studies were excluded from the synthesis of evidence.

### Data extraction and synthesis

Quantitative data were extracted and entered in a spreadsheet (Supplementary Table 2), including information related to study design, characteristics, sample size, intervention characteristics, and results. A narrative synthesis was conducted using an inductive, data-driven, thematic analysis approach (39). The analysis was conducted in a step-wise approach, including familiarization with the data, repeated review of study findings, analysis, and review of themes via an iterative process (40). The key themes were analyzed to examine the study conclusions critically. This process helped synthesize the available evidence through a comprehensive narrative approach surrounded by a critical perspective to ensure evidence-based practice.

## Results

PRISMA guidelines were followed to identify the relevant literature for evidence synthesis. The initial database search using the keywords and MeSH terms described above revealed 503 articles. Among these, 78 articles were duplicates, which were eliminated using the automatic duplication removal tool in EndnoteX9. The next step was screening the remaining 425 articles. Among these, 286 were eliminated due to non-relevance. The eligibility of the remaining 139 articles was verified. Of these, 93 were excluded because they did not fulfill the inclusion criterion, and the reasons for exclusion were mentioned in **Figure 1**. The remaining 46 studies underwent quality assessment. ROB 2.0 and NOS scales were used to assign strength of recommendation for the article’s quality assessment. The 46 studies were categorized as 13 moderate-quality studies and 36 low-quality studies. The thirteen (13) moderate-quality studies, all published in English and available full-text, were included in the review. One out of 13 was a randomized controlled trial, and the remaining 12 non-randomized controlled trials (cross-sectional observational, retrospective designs, qualitative and qualitative exploratory) were mentioned with the origin or country of studies in **Table 1**.

**Table 1:** Characteristics of included studies.

Author (Year)	Origin of study	Sample size Gender (m/f)	Study design	ROB/NOS scores of study quality	Measurement tools/Outcomes	Findings	Limitations
Simon Hatcher et al. (2016) (41)	New Zealand	183 people	RCT	uncertain ROB	Beck Hopelessness Scale	A significant impact of Culturally informed intervention in reducing suicidal behavior and improving outcomes in Māori individuals who presented with intentional self-harm to emergency departments in New Zealand (p=0.03)	The randomization technique used, Self-rated change in score of Beck hopelessness, small sample size, Geographical limitation
Trautmann et al. (2017) (42)	Germany,	253 physicians, 3431 patients data	cross-sectional epidemiological study	6	Primary care physician’s role in the detection, estimation, and treatment of depression	The fact that many primary care patients with depressive disorders are not receiving appropriate treatment underlines the need to improve general practitioners’ diagnosis and treatment choices.	Over-estimation bias, treatment data, and cross-sectional data cannot answer questions on the overestimation of mild to moderate depression
Saini et al. (2014) (43)	UK	336 cases	Retrospective cohort study	5	Key elements of suicide risk assessment in primary care	Depression, suicidal ideation, and a history of self-harm are associated with a high risk of suicide. 25% of primary care spaces had suicide prevention policies, and 33% had staff trained in suicide risk assessments. There is a lack of training in primary care for professionals	Care setting impact, Suicidal ideation impact, Methodological constraint.
McDonnell et al. (2020) (44)	UK	62 professionals	Quasi-experimental	6	Postvention Assisting Those Bereaved by Suicide (PABBS) effectiveness and impact	The training enhanced self-reported understanding, motivation to learn, intention to modify practice, and perceived knowledge, abilities, and confidence. Evidence-based, real-life training materials were highly regarded.	Self-selected sample and reliance on self-report measures are the study’s limitations
Alexandra Pitman et al. (2020) (45)	UK	3193 respondents	Cross-sectional study	6	To test the hypothesis, loneliness relates to post-bereavement suicide attempts and suicidal ideation, even after controlling for network size.	Loneliness was significantly associated with post-bereavement suicide attempts (AOR 1.19; 95% CI 1.14–1.25) and suicidal ideation (AOR 1.24; 95% CI 1.20–1.28) in bereaved adults. No association was found between suicide bereavement and loneliness (adjusted coefficient 0.22; 95% CI - 0.12 to 0.45; p = 0.063). Whether or not participants were suicide bereaved, loneliness increased suicide attempt risk.	Sample predominant with white, females, and highly educated people limits findings to specific geography or setting.
Margot C. van der Burgt et al., (2021) (46)	Netherlands	Interventional region 2586, Control region data=4187	cross-sectional design	6	effectiveness of the SUPRANET program on attitudinal changes in the general public	The results showed that intervention region respondents valued professional help more and were more familiar with the Dutch helpline than control region respondents.	Methodological limitations include self-report bias and time-based questionnaire bias.
Lisa Van Hove et al. (2023) (47)	Belgium	9838 primary caregivers	Cross-sectional	6	Suicide behavior prevalence in 12 or less than 12 years	Passive Suicidal Ideation affected 10.5% of children. Psychiatric, developmental, or behavioral conditions, learning disorders, impulsivity, aggression, and multiple stressful family life events were found to be risk factors for passive SI in childhood.	A cross-sectional study does not determine causality; reports were based on the primary caregiver.
Nicola Clibbens. (2019) (48)	UK	37 patient’s 10/27	Cross-sectional Survey	5	Comprehensive clinical suicide screening Risk measurement tools	Found four themes: 1. Screening for suicidality is culturally appropriate. 2. PH-Q9 questions do not match participants’ lives. 3. Suicide disclosure gives hope to overcome the fear of bad outcomes. 4. Patients talk regarding suicidality is encouraged by listening and caring.	Rating scales’ low suicide risk prediction value limits this approach. By the WHO suicide prevention strategy, the UK recommends a complete clinical risk assessment to identify modifiable suicide risk factors. Substance use, coping, and mental health are modifiable.
Nadia Younes et al. (2020) (49)	France	174 patients	Observational Cross-sectional	6	Primary care at SA and post-SA Data recorded in the Sentinel surveillance system	GPs manage patients at home during SA more frequently (60%), increasing post-SA care. Active follow-up post-SA reduced repeat attempts within 12 months pooled RR = 0.83)	Selection bias. For suicide attempts that did not result in medical intervention, GPs may have forgotten to record SAs or been unaware of them. Second, the Network may alert Sentinel GPs about SAs, preventing them from following French GP protocols. Third, SA management by other prehospital doctors and secondary care management data were not measured, which could affect primary care. The results apply only to non-gatekeeping health systems.
India Bellairs-Walsh et al. (2020) (50)	Australia	10 patients	Qualitative Exploratory	6	GPs’ essential role in youth suicide prevention by engaging in the detection, assessment, and care of youth with suicidal behavior and self-harm	Youth suicide prevention relies on GPs detecting, assessing, and treating suicidal and self-harming behavior. Youth’s readiness to reveal suicide and self-harm risks to GPs depends on knowledgeable, clear, non-judgmental communication and a positive therapeutic relationship.	Small sample size, geographical limitations, Convenience sampling technique, Selection bias, Lack of specific inclusion criteria, and Risk assessment variability.
Elke Elzinga et al. (2020) (51)	Netherland	21 participants: 13 (62%) GPs 5 (24%) MHSS 3 (14%) non-clinical professionals	Qualitative study	6	SUPRANET is perceived as useful for improving suicide prevention practices	SUPRANET enhances suicide prevention. PCPs learned suicide prevention and how to identify suicidal patients through training.	Voluntary participation may attract primary care clinicians interested in suicide prevention. However, the small sample size, inherent sampling bias, restricts the statistical capacity to apply findings to a diverse population. Early adopter syndrome, as everyone wants to be interviewed, also causes the sample size to be small.
Maria Michail al., (2022) (52)	UK	13 GPs	Observational study	5	Role of educational resource in clinical practice Assessing and managing suicide risk among young people Efficient decision-making	Five of 13 GPs said educational materials made them more competent in identifying suicide risk in clinical practice and allowed them to link patients to internet resources.	Small sample size, convenience sampling, geographical evaluation, selection and social desirability bias, interest bias, unknown response rate, and survey questionnaire construct validity were not tested.
Matthew C. Aalsma et al. (2022) (53)	(USA)	200 patients	Retrospective observational study	5	Suicide management within primary care follow-up assessments and referral practices	Follow-up is crucial for adolescents with suicidal ideation after primary care visits, as over 80% of those who attempted suicide had received mental health treatment. [3] Primary care screening for suicidal thoughts and behaviors is crucial for identifying at-risk adolescents (≥50% attend yearly primary care visits).	Limited record resources and incomplete information, physician discretion in interpreting the concern vs. no concern, lack of risk assessment detail, geographical limitation, and limited Clinical Decision Support system access to some clinics. Despite the evaluation given, clinicians applied guidelines differently.

### Risk factors for suicide and suicidal thoughts

Van Hove et al. (2023) identified associations between passive suicidal ideation (SI) in 9-year-olds and prediagnosed psychiatric, developmental, or behavioral conditions, impulsivity, aggression, and stressful family life events. This study emphasized the need for passive SI prevention programs and clinical risk assessments for young children (47).

In adults, loneliness, depression treatment gaps, cultural and social factors were significant risk factors. Pitman et al. (2020) found that higher loneliness scores were associated with post-bereavement suicide attempts and suicidal ideation in 3193 UK bereaved adults. They showed that loneliness after a friend or relative’s sudden death increases suicidal thoughts and attempts, independent of social support. Future longitudinal studies are needed to understand these complex relationships between loneliness, stigma, mental illness, social support, and suicidality in bereaved individuals (45).

Trautmann et al. (2017) analyzed the frequency and type of depressive disorder treatment in German primary healthcare, highlighting the need for better diagnosis and treatment of depressive disorders, as many patients are undertreated (42).

Hatcher et al. (2016) tested a culturally informed intervention in New Zealand, which reduced hopelessness and self-harm recurrence in Māori people hospitalized for intentional self-harm. Over the next year, non-self-harm hospital presentations significantly dropped, highlighting the importance of cultural factors in managing suicidal behavior (41).

These studies collectively underscore the role of loneliness, depressive disorders, and cultural and social factors in contributing to suicidal ideation across different geographical regions. Although each study has its limitations, the significant factors identified highlight the need for targeted interventions to prevent suicide and manage its risk factors effectively.

### Barriers to suicide prevention

The review identified several barriers to effective suicide prevention. Bellairs-Walsh I. (2020) reported barriers such as inadequate information, judgmental communication, absence of positive therapeutic relationships, and insufficient tangible support and resources (50). Aalsma et al. (2022) found inconsistent follow-up issues (53). Saini et al. (2014) indicated variability in risk assessment and poor communication (43). Van der Burgt et al. (2021) examined stigma and negative attitudes as significant barriers to suicide prevention efforts (54). These barriers impede the identification and management of at-risk individuals, highlighting the need for improved communication, support structures, and holistic approaches in primary care settings.

### Suicide prevention strategies

#### Clinical training and continued education

The review identified clinical training programs as essential for preventing suicide. McDonnell et al. (2020) applied the Postvention Assisting Bereaved by Suicide (PABBS) training program in the UK for 62 professionals, finding that it improved clinicians’ knowledge, skills, and confidence in supporting suicide-bereaved parents (44). Michail et al. (2022) indicated that the Royal College of General Practitioners’ educational resource is acceptable and useful in clinical practice, emphasizing the need for ongoing education in youth suicide risk assessment and management (52).

#### Comprehensive clinical assessment

Comprehensive clinical assessments are crucial for identifying and managing modifiable suicide risk factors. Clibbens (2019) interviewed 37 individuals and of those who finished a complete suicide risk assessment, three (21%) had the highest suicide risk score, finding that thorough clinical risk assessments are required to identify modifiable suicide risk factors such as coping strategies, substance abuse, and psychological wellness (48).

#### Primary care management post-suicide attempt

Effective management in primary care following a suicide attempt can reduce the risk of subsequent attempts. Younès et al. (2020) determined that emergency departments provide primary healthcare following a suicide attempt, with GPs managing a third of patients during that period. The study emphasized the need for more post-discharge communication between GPs and ED staff to improve outcomes (49).

#### Holistic risk assessments and collaborative care

Holistic risk assessments and collaborative care are important for preventing youth suicide. Bellairs-Walsh et al. (2020) reported that youth prefer holistic risk assessments, collaborative individualized care, and tangible support and resources from their GPs. Their qualitative exploration revealed that young people’s willingness to disclose suicide and self-harm risks to a GP is facilitated by the GP being adequately informed, clear and non-judgmental communication, and a positive therapeutic relationship (50).

#### Community and program-based interventions

Community-based programs like Suicide Prevention Action Networks (SUPRANET in the Netherlands focus on attitudinal changes in the general public, particularly stigma, taboo, and attitudes toward professional help-seeking. Van der Burgt et al. (2021) provided evidence of the effectiveness of the SUPRANET Community program in reducing stigma and taboo around suicide (54). Elzinga et al. (2020) found that SUPRANET training boosted primary care providers’ awareness and knowledge of suicide prevention, improving their ability to recognize at-risk patients (51). Michail et al. (2022) assessed the effectiveness of RCGP online training in assisting GPs in the assessment and management of suicide risk in consultations with young people, indicating its potential acceptability and benefit in clinical practice (52).

#### National guidelines and policies for primary care practice

Aalsma et al. (2022) emphasized the need for national guidelines to standardize primary care follow-up of adolescents with suicide concerns. The study highlighted variability in reporting depression diagnosis, suicidal ideation, and referrals to behavioral health therapy, emphasizing the importance of consistent follow-up protocols to improve suicide prevention efforts. National standards are essential for consistent and effective management of suicide risk in primary care settings (53).

## Discussion

This review identified various risk factors for suicide among different age groups. In children aged 12 or below, pre-diagnosed psychiatric, developmental, or behavioral conditions, impulsivity, aggression, and stressful family life events were significant risk factors. Among adults, loneliness, depression treatment gaps, and cultural and social factors were identified as major risk factors for suicide and suicidal ideation. These findings are consistent with the meta-analysis conducted by Favril et al. (2022), which reported that clinical factors such as mental health disorders, history of self-harm, family history, sociodemographic factors, and adverse life events are highly associated with suicide attempts and ideation in adults (OD>2) (55).

The review also identified several barriers to effective suicide prevention. These include inadequate information, judgmental communication, absence of a positive therapeutic relationship, inadequate holistic risk assessment, lack of individualized and collaborative care, insufficient tangible support and resources, inconsistent follow-up, variability in risk assessment, poor communication, stigma, and attitudes. These barriers highlight the complexities of treating at-risk patients and underscore the importance of individualized approaches that consider patients’ unique circumstances and help-seeking barriers. For instance, Han et al. (2017) found that help-seeking rates among women were not higher, possibly due to the stigma surrounding suicide and the tendency to hide suicidality (56).

Effective methods to prevent suicide, as reported in the included studies, encompass clinical and educational training programs such as the Postvention Assisting Bereaved by Suicide training program and continued education, comprehensive clinical assessments, holistic risk factor assessments, collaborative and individualized primary healthcare, and community and program-based interventions like cultural interventions and the SUPRANET program. These methods emphasize the necessity for national standards and regulations to update primary care practices for managing patients at suicide risk, as current approaches vary widely, and consistent follow-up strategies are lacking.

Despite these findings, the review is limited by geographical region, small sample size, and study design. These limitations highlight the need for future studies to ensure more robust evidence synthesis, which is crucial for validating preventive interventions in primary care, responding effectively to suicide attempts, and early detection of individuals at risk.

In conclusion, while this review provides valuable insights into the risk factors and barriers to suicide prevention, it also underscores the need for comprehensive assessment and strategies to ease barriers to accessing help. The difficulty of predicting suicide risk is acknowledged as a complex and challenging task, with many contributing factors. However, depression remains a highly significant indicator of suicide, with approximately 2 percent of individuals with severe depression ultimately succumbing to suicide (57), a rate slightly higher than the 1.6 percent of the total U.S. population who die by suicide (58). This complexity indicates that multiple elements need to be considered to explain suicide comprehensively. Therefore, comprehensive assessments and efforts to break down barriers to accessing help are essential for effective suicide prevention.

### Comparison of gatekeeping vs. non-gatekeeping health care systems for suicide prevention efforts

Younès et al. (2020) provided insight into the role of general practitioners (GPs) in caring for patients who have attempted suicide, particularly in the context of gatekeeping versus non-gatekeeping healthcare systems (49). In gatekeeping nations (e.g., the Netherlands, Spain, and the United Kingdom), where patients must be referred to specialists, GPs play a crucial role in managing patients who have attempted suicide. This is different from non-gatekeeping nations (e.g., Belgium, France, Germany, Canada, Switzerland), where patients have direct access to specialists.

In non-gatekeeping countries, GPs are often the primary professional caregivers for a significant proportion of patients who attempt suicide. For example, data from the Belgian Network of Sentinel General Practices indicate that GPs are the primary caregivers for 19.1% of these patients. This is supported by research from Boffin et al. (2015), which shows that 54.2% of patients who have attempted suicide consult with their GPs, particularly in non-gatekeeping areas. However, barriers to GP management of these patients, such as patient refusal of care and lack of trust in GP decision-making, have been noted. These issues reflect broader challenges observed by healthcare staff in similar situations (59). Future research should explore these barriers in more depth to develop effective interventions.

Additionally, the findings revealed that GPs provide primary care for young patients with self-cutting behaviors, while those with self-poisoning are typically hospitalized (60). This study did not differentiate between patients who remained at home and those referred to hospitals, likely due to small sample sizes.

Houston et al. investigated follow-up care after a suicide attempt, emphasizing the critical role of primary care. In regions with gatekeeping systems, GPs are frequently involved in post-attempt management (61). However, in non-gatekeeping nations like Canada, patients often do not receive follow-up treatment after a suicide attempt due to the healthcare system structure. Their findings highlight a significant gap in post-suicide attempt care, suggesting that brief contact treatments after emergency department (ED) discharge can successfully prevent subsequent suicidal crises (62, 63). Notably, patients are more likely to receive follow-up care from GPs if the GP was the initial post-suicide attempt care provider, indicating the importance of strong physician-patient-family collaboration (64, 65).

In summary, Younès et al. (2020) illuminate the vital role of GPs in managing patients who have attempted suicide across different healthcare systems (49). Despite the important role of GPs, challenges such as patient rejection and gaps in post-suicide attempt follow-up care persist. Enhancing the physician-patient-family relationship, strengthening connections between GPs and ED staff, and implementing brief contact treatments after ED discharge are critical strategies to improve care quality for these patients. Addressing barriers that prevent individuals, especially women, from seeking help is essential to ensure at-risk patients receive the necessary support. Future research on the multifaceted experiences of GPs and patient perspectives on GP care will be crucial in developing targeted interventions to improve outcomes and reduce suicide risk.

### Integrating behavioral health with multimodal communication to address patient and home safety measures

Hunter et al. observed higher rates of referral to behavioral health therapy compared with conversations about weapon availability and safety planning, indicating a potential priority imbalance. While mental health referrals are crucial, this should not supplant critical discussions about patient safety at home (66). The perception of suicidal patients as potential liabilities by physicians may contribute to this imbalance, revealing systemic difficulties in healthcare delivery and patient management. To protect the well-being of patients, particularly those at risk for suicide, primary care clinicians must feel empowered and supported in addressing patient weapon access and safety planning.

The findings highlight significant gaps in primary care regarding weapon availability and safety planning procedures. These gaps, potentially influenced by liability and malpractice concerns, underscore the need for systemic reforms that prioritize patient safety. A multimodal strategy is required to address these concerns, encompassing professional education and support, clear rules and processes, and a cultural shift to prioritize discussions of patient safety. Bridging these gaps will enable clinicians to better fulfill their responsibilities in safeguarding and promoting patient well-being, especially among patients at risk for suicide.

### Role of holistic psychosocial assessment and GP training in suicide prevention initiatives

Bellairs-Walsh et al. (2020) (50), Hawgood and De Leo (2016) (67) highlighted the significant role of GPs in youth suicide prevention through the assessment, detection, and care of those with suicidal behaviors and self-harm (68). The emphasis on holistic, psychosocial-based assessments aligns with best practices and principles of youth-friendly services, prioritizing collaboration and individualized care that reflects young people’s preferences (69). This study’s exploration of youth preferences regarding risk conceptualization adds a valuable dimension to the literature, emphasizing the need for comprehensive, collaborative care.

The authors also underscored the importance of positive therapeutic encounters with healthcare services for young people (70). GP interpersonal skills are vital to suicide prevention initiatives, highlighting the necessity for empathetic approaches (71). While the use of Clinical Decision Support System (CDSS) technologies by GPs to aid in identifying and treating patients at suicide risk is feasible, the real-world usefulness and implementation issues of these tools require further investigation. Another relevant proposal is for GP training programs to improve communication with young patients exhibiting suicidal behaviors and self-harm. Effective communication and successful interventions and support are key to creating trust and rapport with young people (72).

Finally, this study revealed young people’s preferences within the discourse on suicidality and self-harm. Guidelines for patient-centered language, holistic evaluations, and GP training may improve suicide prevention initiatives. Future research should evaluate the efficacy of CDSS tools and the development and implementation of GP training tailored to work with at-risk adolescents. Overall, these studies lay the groundwork for enhancing care quality and support for young people experiencing suicidal ideation and self-harm.

### Strengths

This review adheres to PRISMA guidelines and employs Risk of Bias 2.0 and the Newcastle Ottawa scale for quality assessments. It identifies significant suicide risk factors and barriers to prevention among children and adults.

The review suggests clinical, educational, community, social, and cultural strategies to address these barriers. It emphasizes comprehensive clinical assessments and the acceptability of suicide screening in primary care with empathetic healthcare.

Sentinel Network data supports thorough suicide attempt (SA) assessments and highlights the importance of follow-up in primary care. The role of GPs in adolescent suicide prevention underscores clear communication and strong therapeutic connections, noting that recording mental health information may affect young people’s help-seeking behavior.

The SUPRANET program insights emphasize the importance of including PCPs in suicide prevention, showing that their training improves awareness and understanding.

The review also examines the RCGP online education resource, noting its early positive impacts on suicide risk assessment and management in primary care. This resource aids in follow-up treatments and referrals for youths with suicidal thoughts, potentially reducing their risks.

### Limitations

This review has several limitations. It included only one randomized controlled trial and 12 non-randomized controlled trials, all with small sample sizes. This reduces the statistical power of the findings and may limit their generalizability. Additionally, the patient health questionnaire-9 has a relatively low predictive value for assessing suicide risk, so the results were interpreted with caution during the methodological assessment.

The Sentinel Network data used in some studies have limitations as well, such as GPs underreporting suicide attempts and potential selection bias, which may further limit the generalizability of the findings. The included studies were also regional examinations, which restrict their broader relevance. Other shortcomings include the reliance on medical records, which may not capture all treatment and follow-up beyond primary care settings and carry the potential for clinician subjective interpretation.

Furthermore, future studies would benefit from more diverse samples, as most reviewed studies focused predominantly on Black females or white males. This lack of diversity underscores the importance of using caution when interpreting the study findings. It also provides opportunities for further research to develop more inclusive and representative suicide prevention measures in primary care settings.

Suicide is an extraordinarily complex phenomenon and predicting it can be incredibly challenging. Many factors contribute to suicidal thoughts and behaviors, making it difficult to pinpoint specific causes or predict outcomes with certainty. This complexity is compounded by the unique circumstances and mental health conditions of each individual. Comprehensive assessments and personalized approaches are therefore crucial in effectively managing suicide risk.

The inherent difficulty in predicting suicide highlights the need for robust, evidence-based strategies and continuous improvement in clinical practices. By understanding and addressing these limitations, future research can contribute to more accurate risk assessments and effective prevention measures.

In summary, while this review provides valuable insights, the highlighted limitations necessitate cautious interpretation of the findings. They also emphasize the need for continued research to refine and develop suicide prevention strategies that are inclusive, reliable, and effective in diverse primary care settings (**Table 1**).

### Clinical implications

The review primarily included non-randomized trials, with only one randomized controlled trial (RCT). The strength of the evidence was moderate, which limits its direct translation into practice. Nevertheless, the findings underscore the importance of comprehensive clinical assessments, which involve evaluating factors influencing patient diagnosis, managing risk factors, improving clinical assessments, and actively listening to patients. These strategies are crucial for reducing suicide rates and should be applied in clinical settings.

Training courses that encourage physicians and specialists to implement timely, effective, and efficient interventions with at-risk patients are also essential. Incorporating these techniques into national practice guidelines can ensure evidence-based practice, thereby improving the quality of care for individuals at risk of suicide.

### Future recommendations

Despite the moderate quality of the evidence, there is an immediate need for collaboration among researchers, clinicians, and policymakers to facilitate future research, translate these findings into practice, and devise standardized protocols or national guidelines. These efforts would ensure the effective implementation of evidence-based suicide prevention strategies in primary care settings. Future research should focus on conducting prospective or comparative intervention trials with comprehensive, rigorous methods that ensure sample diversity, thereby improving the reliability and generalizability of the findings.

Expanding our understanding of suicide prevention measures in primary care settings is also crucial. It is important to understand how physicians estimate suicidality risk to build more effective risk assessment processes and therapies. Additionally, future studies should capture the contributions of other healthcare practitioners, including social workers and mental health experts, in managing patient suicide risk, as they can significantly influence patient outcomes.

In conclusion, while the current evidence provides valuable insights, further research with more robust methodologies is needed to enhance the clinical management of suicide risk. Collaborative efforts will be essential in developing effective, standardized approaches to suicide prevention in primary care settings.

## Conclusion

This review identified significant risk factors, barriers, and effective strategies for suicide prevention among children and adults in primary healthcare settings. For children under 12, risk factors included preexisting psychiatric, developmental, or behavioral disorders, impulsive behaviors, aggressiveness, and significant family stress. For adults, key risk factors were loneliness, gaps in depression treatment, and cultural and social factors.

Barriers to effective prevention included insufficient information, judgmental communication, lack of a positive therapeutic relationship, inadequate holistic risk assessments, lack of individualized care, insufficient support and resources, inconsistent follow-up, variability in risk assessment, poor communication, stigma, and negative attitudes. Addressing these barriers is crucial for improving suicide prevention efforts.

Effective strategies identified include the Postvention Assisting Bereaved by Suicide training, continued education, comprehensive clinical assessments, holistic risk factor assessments, collaborative primary healthcare, and community-based interventions like the SUPRANET program. These strategies emphasize the importance of identifying modifiable risk variables, such as substance use, coping techniques, and psychological well-being.

The complexity of identifying risk factors underscores the need for rigorous risk assessment processes and a holistic approach, considering the benefits and limitations of different healthcare systems. GP engagement following a suicide attempt significantly influences post-attempt outcomes, highlighting the crucial role of primary care in suicide prevention.

Successful initiatives include comprehensive risk assessments, collaborative treatment, and concrete support for GPs. Youth-friendly practices in primary care, such as those promoted by the SUPRANET program and RCGP educational resources, improve prevention practices. The absence of national standards for primary care follow-up with adolescents with suicidal ideation highlights the need for development in this area.

In summary, improving comprehensive evaluations, primary care engagement, youth-friendly practices, useful tools and resources, and establishing national standards are critical to enhancing primary care suicide prevention efforts.

## Data Availability

The original contributions presented in the study are included in the article/**Supplementary Material**. Further inquiries can be directed to the corresponding author.
